# Total Knee Arthroplasty for Severe Crystalline-Induced Arthropathy: A Case Report From a Third-Level Hospital

**DOI:** 10.7759/cureus.82462

**Published:** 2025-04-17

**Authors:** Diego Hernández-Penagos, Jose Melesio Hilera-Camara, Carlos David Franco-Gonzalez, Juan Jose Serrato-Rodriguez, Jose Luis Tejero-Lopez, Francisco Armando Vera-Aviles

**Affiliations:** 1 Orthopedic Surgery, Hospital Regional Elvia Carrillo Puerto, Institute for Social Security and Services for State Workers (ISSSTE) Facultad de Medicina de la Universidad Autónoma de Yucatán, Merida, MEX; 2 Clinical Sciences, Universidad Marista de Mérida, Merida, MEX; 3 Orthopedics and Traumatology, Hospital Regional Elvia Carrillo Puerto, Institute for Social Security and Services for State Workers (ISSSTE) Facultad de Medicina de la Universidad Autónoma de Yucatán, Merida, MEX

**Keywords:** arthroplasty, gout disease, joint replacement surgeon, knee arthroplasty, knee replaecment

## Abstract

Gout is an inflammatory arthritis caused by monosodium urate crystal deposition, leading to progressive joint destruction and functional impairment. While pharmacologic treatment remains the standard, advanced cases with intra-articular and intraosseous tophi may require surgical intervention. Total knee arthroplasty (TKA) has been reported as a viable option for managing severe tophaceous gout with structural bone defects, improving joint function and pain control when conservative therapy fails. We present the case of a 56-year-old male with a long-standing history of gout and progressive knee pain refractory to medical treatment. Imaging revealed extensive intraosseous tophi with cavitary bone defects, prompting the decision for TKA with bone allograft reconstruction. The patient experienced favorable postoperative recovery, demonstrating significant improvement in joint mobility and function, with no complications observed. This case highlights the role of surgical intervention in the management of advanced tophaceous gout as part of an integrated approach alongside pharmacologic control.

## Introduction

Gout is an inflammatory arthritis caused by the crystallization of monosodium urate (MSU) due to chronic hyperuricemia. It is the most common form of inflammatory arthritis worldwide and is associated with metabolic disorders such as obesity, hypertension, diabetes mellitus, and chronic kidney disease. The disease follows a progressive course, beginning with acute attacks of arthritis and evolving into chronic tophaceous gout when urate crystal deposition persists, leading to severe joint damage and disability [1-5].

In advanced stages, intra-articular and extra-articular tophi develop, contributing to joint instability, subchondral bone erosion, and cavitary defects. These complications are particularly debilitating when weight-bearing joints such as the knee are affected. Persistent inflammation due to tophaceous deposits can accelerate joint destruction, increase the risk of secondary osteoarthritis, and significantly impact patient mobility and quality of life [6-9].

Pharmacologic therapy remains the first-line treatment for gout, aiming to reduce serum urate levels and prevent disease progression. However, a subset of patients develops refractory disease, characterized by persistent tophi and progressive joint deterioration despite medical treatment [3,10,11]. In these cases, surgical intervention becomes necessary to restore joint function and relieve pain. Surgical options include arthroscopic debridement, synovectomy, tophi excision, and, in cases of severe joint destruction, total knee arthroplasty (TKA) [12-14].

TKA is considered the treatment of choice for end-stage gouty arthritis when extensive joint damage leads to severe pain and functional limitations. However, challenges such as bone loss, soft tissue involvement, and a higher risk of postoperative complications must be considered. Despite these concerns, studies have reported favorable outcomes in patients undergoing TKA for advanced gout, demonstrating significant improvements in pain relief, range of motion, and overall functionality [15].

We present the case of a 56-year-old male patient with chronic tophaceous gout affecting the right knee, who experienced severe pain and functional impairment despite pharmacologic management. Given the presence of extensive intra-articular and intraosseous tophaceous deposits, TKA with bone allograft (BA) reconstruction was performed, achieving a successful clinical outcome. This report underscores the role of surgical management as part of a comprehensive approach for advanced gouty arthritis, particularly in cases where joint destruction compromises function and quality of life.

## Case presentation

A 56-year-old male with a 10-year history of gout presented with right knee pain. His body mass index (BMI) was 26, and no other significant comorbidities were reported. His surgical history was notable for an arthroscopic procedure in 2014 and an open synovectomy on the same joint in 2015. Despite medical management, the patient experienced progressively worsening pain and functional limitations that interfered with his daily activities. As part of the preoperative optimization, he was counseled on weight management and initiated on a physical therapy regimen to enhance muscle strength.

**Figure 1:**
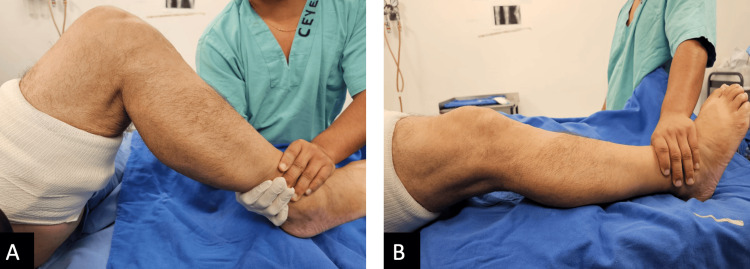
Preoperative range of motion of the right knee A: maximum flexion of the right knee in lateral view: 85º; B: maximum extension of the right knee, showing a flexion contracture of 30º.

**Figure 2:**
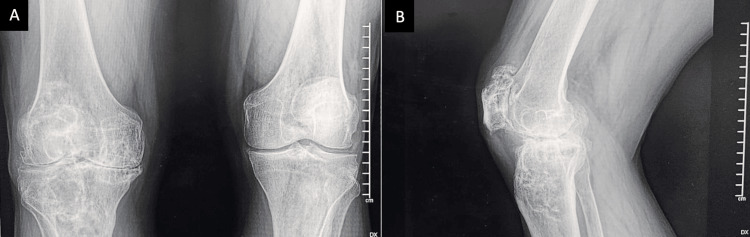
Radiographic images of the right knee A: anteroposterior; B: lateral projections show a bilateral reduction in joint space with varus deformity. Marginal lytic bone lesions are noted at the subchondral level of the proximal tibia and distal femur, exhibiting well-defined sclerotic edges and no periosteal reaction. At the patellar level, there is a reduction in joint space along with marginal osteophytes present at the upper pole.

On physical examination, an anterior scar on the right knee was noted, along with varus deformity. There was tenderness upon palpation along the medial joint line. The right knee exhibited a flexion contracture of 10°, with active flexion limited to 80° (Figure 1). Distal neurovascular examination was intact. Imaging studies, including anteroposterior and lateral X-rays of the right knee, showed decreased medial joint space, bilateral subchondral sclerosis of the tibial plateaus, well-defined lytic lesions in the tibial and femoral epiphyses compatible with intraosseous tophi, and multiple marginal osteophytes (Figure 2).

**Figure 3:**
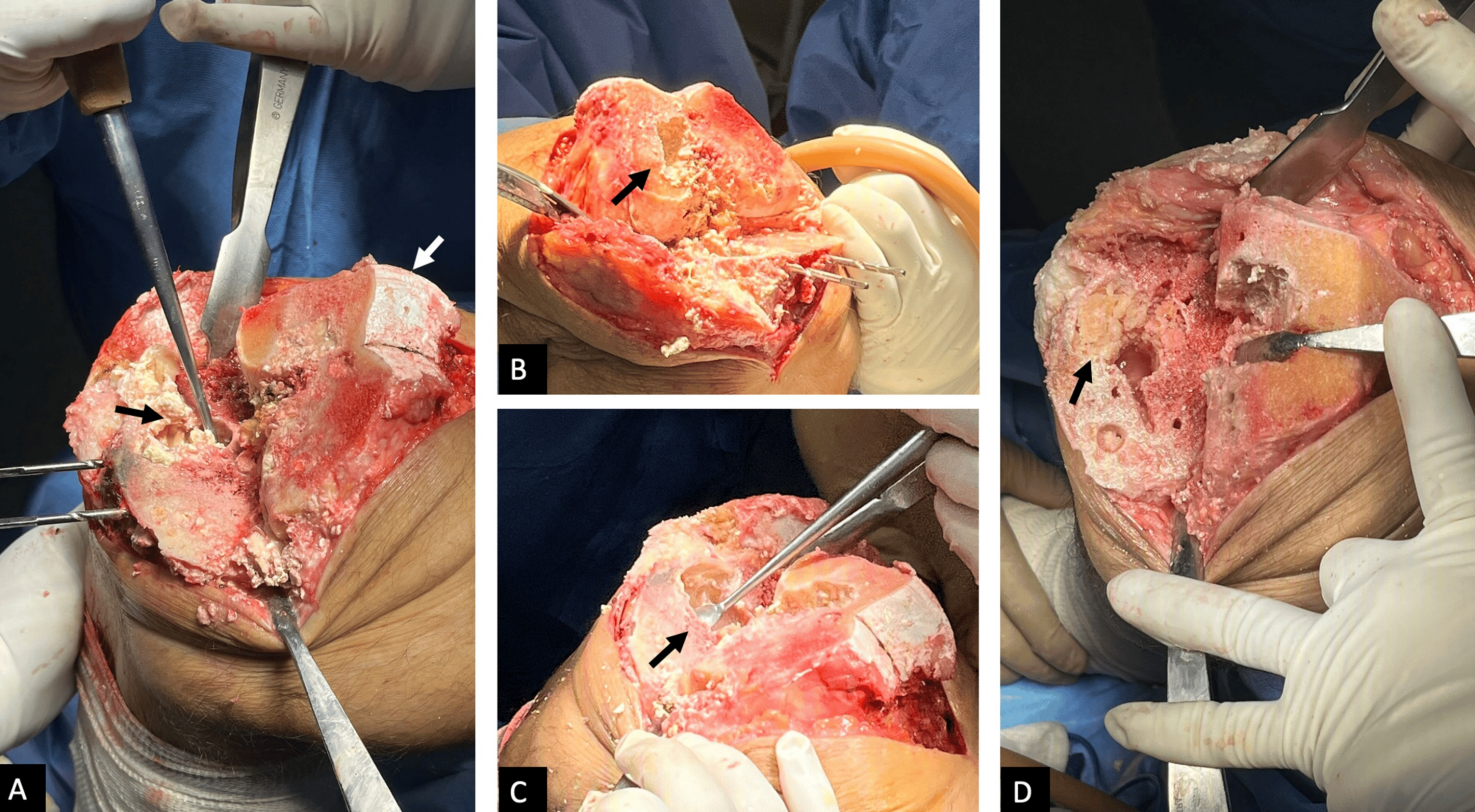
Intraoperative photograph of the right knee A: significant infiltration of the femoral articular cartilage (white arrow) by tophaceous urate deposits is observed, along with a cavitary bone lesion in the subchondral bone of the proximal right tibia (black arrow) filled with semi-solid yellowish tissue; B: cavitary bone lesion in the subchondral region of the right lateral condyle prior to anterior and posterior femoral cuts (black arrow); C: full extent, depth, and diameter of the cavitary lesion in the subchondral bone of the proximal tibia and distal femur (black arrow); D: filling of the cavitary bone defect in the proximal tibia with bone allograft chips and preparation for the placement of prosthetic implants (black arrow).

Following preoperative evaluation by internal medicine and anesthesiology departments, the patient underwent surgery for pain management and functional restoration using a medial parapatellar approach following the Insall technique. A posterior-stabilized cemented TKA was performed. Intraoperatively, a cavitary defect measuring approximately 1 cm in diameter and 8 mm in depth was identified in the lateral femoral condyle. Additionally, another cavitary defect, approximately 1.5 cm in diameter and 6 mm in depth, containing semi-firm yellowish tissue, was observed in the proximal tibia. This tissue was removed and cleaned with pulsatile irrigation with saline solution with antibiotics (Figure 3). Pathological examination confirmed the presence of gouty tophi.

**Figure 4:**
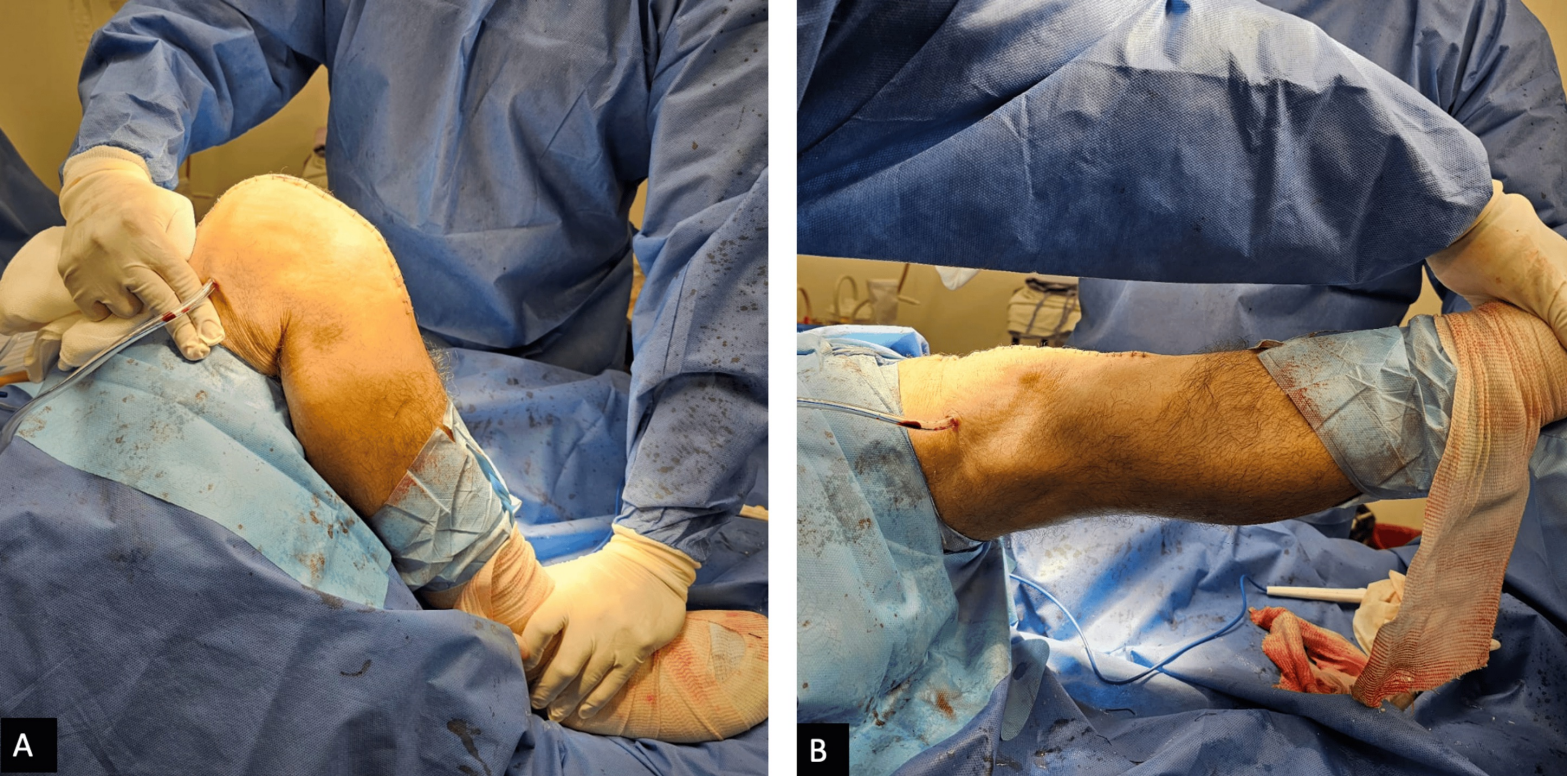
Intraoperative photograph of the angular correction and range of motion improvement A: maximum flexion of the right knee in lateral view: 110º; B: maximum extension of the right knee: 0º.

Before placing the definitive implants, surgical lavage was performed with 3 liters of saline solution, and the cavitary defects in the femur and tibia were filled with 30 cc of freeze-dried cancellous BA in chips. Angular correction and improvement in range of motion were achieved during the surgical procedure (Figure 4). 

**Figure 5:**
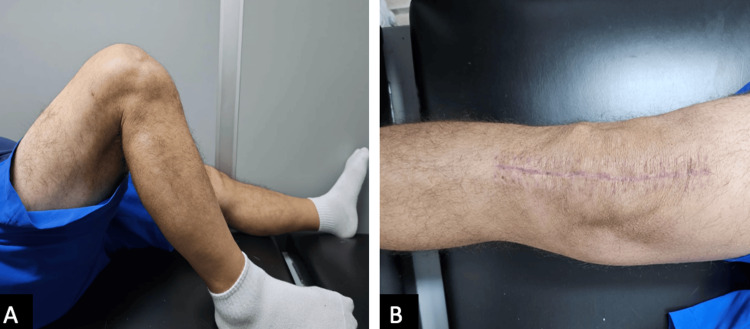
Clinical photograph of angular correction and range of motion improvement six weeks post-surgery A: maximum flexion of the right knee in lateral view: 110º; B: maximum extension of the right knee: 0º, and close surgical wound after six weeks post-surgery.

**Figure 6:**
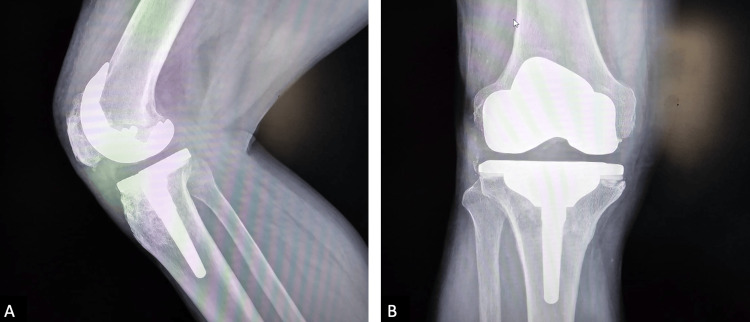
Postoperative X-ray of the right knee joint A: anteroposterior; B: lateral view, and the bone allograft appeared to be intact with minimal remodeling.

The patient was discharged on the second postoperative day with instructions for ambulation using a walker and total support. Postoperative clinical follow-up included evaluations at six weeks, three months, six months, and one year. The Knee Society scoring system was utilized for clinical and functional assessment one year post-surgery, yielding a score of 85 (excellent) (Figures 5, 6). During hospitalization, no specific anxiety or stress-related interventions were required. No complications occurred, and no additional medication beyond standard pain control and anti-inflammatory treatment was necessary. The average postoperative range of motion at the final follow-up was 110°, indicating significant improvement. The patient was referred to physical therapy to improve ambulation and muscle strength.

## Discussion

Gout is an inflammatory arthritis caused by the accumulation of MSU crystals in joints and surrounding tissues, leading to episodes of acute inflammation and progressive joint destruction. This crystal accumulation results from chronic hyperuricemia, which can arise from either overproduction or reduced excretion of uric acid. Risk factors such as obesity, hypertension, chronic kidney disease, and diuretic use increase the likelihood of developing the disease. In advanced stages, tophi form visible deposits of crystals surrounded by inflammatory tissue that affect both soft tissues and underlying bones, commonly involving joints such as the first metatarsophalangeal joint, ankle, elbow, and knee, although any joint can be affected over time. Tophi are characterized by the development of lytic lesions and cavitary defects, contributing to joint instability and loss of function [2].

TKA is the recommended surgical treatment for severe joint damage that impairs patient function and quality of life, especially when pharmacologic options fail to halt disease progression. This procedure replaces the affected joint surfaces with prosthetic components, restoring mobility and alleviating chronic pain. In patients with advanced gout, the accumulation of intra-articular and intraosseous tophi leads to deformities and cavitary defects, posing additional challenges for implant placement. BAs are particularly important in these cases to fill the structural defects caused by tophaceous bone destruction. BA improves implant stability and promotes adequate integration, reducing the risk of implant loosening or mechanical failure over the long term. Chernoff et al. and George et al. agree that TKA is a reasonable intervention for patients with significant joint damage unresponsive to pharmacological treatment [3,8]. TKA allows for the restoration of joint alignment, the correction of deformities, the elimination of bone defects, and improvement in function, providing relief from chronic pain. Furthermore, the use of BA is essential to address the structural defects caused by tophaceous infiltration, ensuring implant stability and optimizing long-term integration [3,5,10].

Complications have been reported in patients undergoing TKA for tophaceous arthritis, according to Chernoff et al. and George et al. [3,8]. Both authors emphasize that periprosthetic infections are the most common complications and pose a diagnostic challenge since symptoms such as pain, inflammation, and erythema mimic those of an acute gout flare. In this patient, no febrile episodes or other signs of periprosthetic infection were observed, suggesting successful surgical management, resulting in an uncomplicated recovery [3,4,9].

In the present case, the patient achieved a range of motion of 110° and a Knee Society Score of 85 at six months postoperatively. The absence of complications and significant recovery further demonstrates the effectiveness of TKA in patients with advanced joint damage. These results are consistent with those reported by Chernoff et al., who documented significant improvements in mobility and quality of life following TKA in cases of severe gout-related arthropathy [3].

Follow-up is essential to evaluate clinical and functional improvement, ensure pain relief, and monitor for signs of infection, which is a major concern in patients with gout. Insufficient control of serum uric acid levels may compromise outcomes and lead to new joint complications. For this reason, the patient was referred to rheumatology to ensure optimal management of serum uric acid levels. Regular assessment of the BA and postoperative rehabilitation are critical to ensure successful integration and prolong the performance of the implant [11].

## Conclusions

This case highlights the role of surgical intervention in the management of advanced tophaceous gout as part of an integrated approach alongside pharmacologic control. TKA not only eliminates intra-articular crystalline deposits but also corrects deformities and provides mechanical stability, enhancing joint mobility and range of motion, which in turn significantly improves the patient’s quality of life.
